# Recombinant TSH Performs as Well as Thyroid Hormone Withdrawal for Iodine-131 Therapy With Dosimetry for Thyroid Cancer

**DOI:** 10.1210/jendso/bvaf050

**Published:** 2025-03-18

**Authors:** Anupam Kotwal, Abbey Fingeret, Jarod Hamsa, Dana Awad, Craig Johnson, Frank Rutar, Carrie Carson, Anery Patel, Whitney Goldner

**Affiliations:** Division of Diabetes, Endocrinology and Metabolism, Department of Internal Medicine, University of Nebraska Medical Center, Omaha, NE 68198, USA; Division of Surgical Oncology, Department of Surgery, University of Nebraska Medical Center, Omaha, NE 68198, USA; College of Medicine, University of Nebraska Medical Center, Omaha, NE 68198, USA; Division of Diabetes, Endocrinology and Metabolism, Department of Internal Medicine, University of Nebraska Medical Center, Omaha, NE 68198, USA; Department of Radiology, University of Nebraska Medical Center, Omaha, NE 68198, USA; Department of Environmental Health and Safety, University of Nebraska Medical Center, Omaha, NE 68198, USA; Department of Environmental Health and Safety, University of Nebraska Medical Center, Omaha, NE 68198, USA; Division of Diabetes, Endocrinology and Metabolism, Department of Internal Medicine, University of Nebraska Medical Center, Omaha, NE 68198, USA; Division of Diabetes, Endocrinology and Metabolism, Department of Internal Medicine, University of Nebraska Medical Center, Omaha, NE 68198, USA; Division of Endocrinology, Metabolism, and Diabetes, Department of Internal Medicine, University of Colorado Anschutz Medical Campus, Aurora, CO 80045, USA

**Keywords:** radioactive iodine, radioiodine, differentiated thyroid cancer, nuclear medicine

## Abstract

**Introduction:**

Dosimetry helps calculate the optimal iodine-131 (I-131) dose for treating metastatic differentiated thyroid cancer (DTC). We aimed to evaluate if recombinant human TSH (rhTSH) and thyroid hormone withdrawal (THW) are equivalent methods of preparation for dosimetry-guided I-131 therapy in metastatic DTC.

**Methods:**

We performed a retrospective cohort study of 51 adults with metastatic DTC who received I-131 with dosimetry from 2010 through 2022. Gamma camera and blood activity measurements were taken following the pretherapeutic I-131 dose. Statistical analysis compared rhTSH and THW groups; *P* < .05 was considered significant.

**Results:**

Fifty-one adults undergoing 55 I-131 dosimetry-guided treatments were included: 22 by rhTSH and 33 by THW. The median age was lower (*P* = .0008), and the proportion of stage IV (*P* = .009) was higher in rhTSH compared to the THW group. The terminal effective half-life at 24 to 48 hours in the whole body was longer in rhTSH compared to THW group (21.9 vs 17.1 hours; *P* = .014), but this difference was less significant when limited to the n = 37 metastatic cases (*P* = .046) and not different for red marrow effective half-life. The calculated allowed I-131 dose was lower in rhTSH compared to THW group (187.5 mCi vs 259.9 mCi; *P* = .0000). Thyroglobulin was higher during treatment in the rhTSH group (*P* = .031), whereas its reduction at 3 months was not different after adjusting for age and stage.

**Conclusion:**

rhTSH is noninferior to THW in preparation for I-131 dosimetry. Compared to THW, rhTSH results in lower calculated allowed I-131 dose after dosimetry, which could translate to fewer side effects or impact on quality of life.

Management of patients with advanced or metastatic differentiated thyroid cancer (DTC) consists of thyroidectomy with or without lymph node dissection, as appropriate, followed by therapy with radioiodine (RAI) iodine-131 (I-131) [1-3]. During RAI treatment, increased TSH is necessary to maximize selective RAI uptake by normal thyroid or neoplastic cells. This TSH increase can be generally obtained by thyroid hormone withdrawal (THW) or recombinant human TSH (rhTSH) administration [1-3]. For metastatic DTC, classically THW has been used with withdrawal for 4 to 6 weeks to increase TSH as the use of rhTSH in this scenario is not approved by the US Federal Drug Administration or European Medicines Agency for this indication, instead has been used based on the Thyrogen Compassionate Use Program. Current national and international guidelines suggest rhTSH be considered in patients with contraindications to THW [1-3].

Like other centers specialized in managing thyroid cancer, rhTSH is used at our institution as a compassionate alternative to THW for RAI therapy using dosimetry for patients with metastatic DTC in whom prolonged hypothyroidism is deemed clinically unsafe or intolerable. Another rationale for using rhTSH is the lower total body, bone marrow, and gastrointestinal radiation exposure for a given administered activity [4]. This practice pattern is supported by studies demonstrating that rhTSH preparation is safe and successful for inducing I-131 uptake in local and metastatic DTC [5-9]. However, a prior study raised the concern for lower RAI uptake in metastatic DTC lesions after rhTSH compared to THW [10]. Additionally, although some dosimetry-guided I-131 studies [5, 7] showed promising results of rhTSH in dosimetry-guided I-131 treatment for metastatic DTC, they did not compare in detail to THW. We expect some discrepancies in the reported efficacy of rhTSH to be explained by a lack of consistent dosimetry in included patients. We hypothesized that rhTSH and THW are equivalent methods of preparation for I-131 therapy using dosimetry for metastatic DTC.

## Materials and Methods

### Study Design

We performed a single-center retrospective cohort study of 51 adult patients with metastatic DTC who received dosimetry-guided I-131 therapy from 2010 through 2022. Patients with end-stage renal disease receiving dialysis were excluded. Treatment decisions were determined during the multidisciplinary endocrine tumor board at our institution. The institutional review board approved this study for human use. Clinical and pathologic variables were collected including patient sex, age, race or ethnicity, body mass index, date of initial cancer diagnosis, extent of surgery, histologic subtype, cancer stage with American Joint Cancer Commission (AJCC) 7th and 8th edition, presence and site of distant metastases, date of I-131 treatment, I-131 dose administered, number of I-131 treatments, cumulative I-131 dose, method of preparation with TWH or rhTSH, pretreatment, treatment, and posttreatment laboratory values, American Thyroid Association risk of recurrence and response to therapy and mortality.

### I-131 Dosimetry Protocol

**Figure 1.**
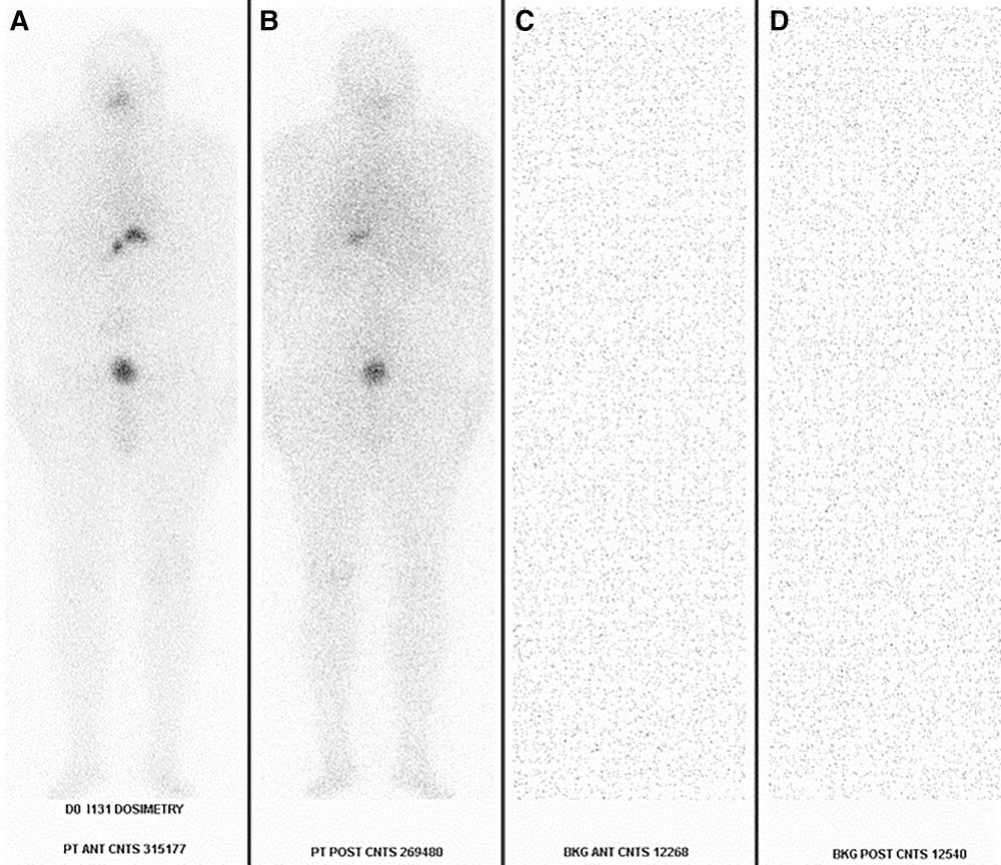
A 2-millicurie (mCi) capsule dose of I-131 was administered to the patient on day 0 of the recombinant human thyrotropin (rhTSH) dosimetry protocol. Laboratory blood draws, whole-body gamma camera imaging of the patient in anterior (A) and posterior (B) projections, as well as background correction gamma camera imaging in anterior (C) and posterior (D) projections were obtained for dosimetry calculations.

A total of 55 I-131 dosimetry protocol treatments were performed in 51 unique patients with biopsy-proven advanced DTC. The patient should not have received recent iodinated contrast, thyroid medications, or supplements that could diminish I-131 uptake. Childbearing-aged women (ages 11-60 years), unless surgically sterilized, must have a negative serum pregnancy test done 3 days before day 0 of the dosimetry protocol. A low iodine diet for 2 weeks before the I-131 treatment dose and 48 hours after the I-131 treatment dose was instituted in both groups. The patient was maintained as nil per oral 4 hours before receiving the I-131 treatment dose and 2 hours after. Patients in the rhTSH group were given 2 subsequent 0.9-mg intramuscular injections of Thyrogen (thyrotropin alfa) on day 0 and day 1 of the dosimetry protocol. Patients in the THW protocol discontinued T3 supplementation for 2 weeks and T4 for 4 to 6 weeks before day 0 of the dosimetry protocol. A background blood draw was obtained, and a 2-millicurie (mCi) dose of I-131 was administered to the patient. Two hours after administration, another background blood draw and whole-body gamma camera imaging of the patient and background were obtained. Then 24-hour and 48-hour laboratory testing of TSH, thyroglobulin (Tg), thyroglobulin antibody (TgAb), and whole-body gamma camera imaging of the patient and background were performed (Fig. 1). Packard COBRA gamma counter was used to assess physiologic clearance of radiotracer from the blood over time. Gamma camera counts and time from I-131 capsule administration were utilized to calculate the maximum I-131 treatment dose allowed by dosimetry.

### Calculation of Maximum Permissible I-131 Dosage

When determining the therapeutic dosage of I-131 to be administered to patients with thyroid carcinoma, the treating physician must know the maximum dose allowable to prevent toxicity. The following guidance from the literature was used to avoid toxicity from I-131 treatment of patients with thyroid carcinoma: (1) body retention of I-131 at 48 hours postadministration should be <120 mCi if no lung metastases are present. If lung metastases are known, the body retention at 48 hours should be < 80 mCi; (2) absorbed radiation dose to blood (red bone marrow) should be <200 rad.

The data needed to perform these calculations were obtained by administering a dosimetry amount of 2 mCi I-131 to the patient and performing whole-body gamma camera imaging scans. Blood draws were undertaken at 2-, 24-, and 48-hour time points after dosimetry administration. To determine the radioactive concentration in blood, 1 mL of the blood draw was pipetted into a scintillation tube and counted in a Packard Cobra Gamma Counter (3″ NaI detector). The predetermined counter efficiency of 48% for I-131 gives the blood concentration of the administered radioactive dose (µCi/mL). The conjugate of the net posterior and anterior gamma camera counts was used for the whole-body retention on imaging.

### Calculation based on Whole Body Retention

The whole-body count measured 2 hours after the dosimetry administration is assumed to be 100% of the administered dose. The fraction of radioactivity retained after 48 hours is a ratio of the 48 hr whole body count to the 2-hour count (Fig. 1). The maximum allowable therapeutic dose of I-131 to prevent toxicity is based upon whole-body retention of I-131 not exceeding 120 mCi (no lung metastases) or 80 mCi (lung metastases known) 48 hours after I-131 administration as follows:


$$Maximum\;Tx\;Dose\;(mCi)=\frac{120\;mCi\;(No\;lung\;mets)\;or\;80\;mCi\;(lung\;mets)}{WB\;Fraction_{48\;hr}}$$

### Calculation based on Red Bone Marrow Dose

The maximum therapeutic dose of I-131 must result in <200 rad to the red bone marrow (RBM). In 2012, our institution changed the RBM dose calculation methodology [11-13]. The maximum therapeutic dose of I-131 must result in a dose < 200 rad to the red bone marrow (RBM). Two different methods were utilized for calculation of the RBM dose, both using the patient's blood concentration of administered I-131. The radiation dose to RBM was calculated using Organ Level INternal Dose Assessment (OLINDA-EXM) version 1.0 software (copyright Vanderbilt University). OLINDA-EXM 1.0 performs internal dose calculations, principally for radiopharmaceuticals, using the RADAR method of dose calculations and RADAR dose factors. RADAR is the Radiation Dose Assessment Resource, which is a working group that maintains resources for internal and external dose calculations, mostly given on a website (www.doseinfo-radar.com), but also in several open literature publications. The OLINDA-EXM 1.0 implements the dose factors from the RADAR web site in a code that permits users to enter kinetic data for radiopharmaceuticals. To calculate the RBM dose, the cumulated activity (µCi-hrs) for the RBM following administration of the dosimetry administration of I-131 was calculated and the patient's blood concentration was used as a surrogate for the RBM. This cumulated activity can be calculated by calculating the total activity (eg, µCi) in the RBM, which is simply the blood concentration of the estimated RBM volume in the patient (using the patient's mass and the RBM volume in either Standard Man or Woman in ICRP Publication 70) and then finding the area under the curve for RBM activity over time after administration.

In 2012, our institution changed the RBM dose calculation using the methodology in which the RBM dose is calculated using the following equation [11]:


$$\begin{array}{rl} D_{RM}(rad/mCi)= & 0.313\times RMBLR\times C_{blood\;}+0.456\\ & \times {\tilde{A}}_{TB}/m_{TB} \end{array}$$

D_RM_ = Dose to red marrow (rad per mCi administered)

RMBLR = Red marrow to blood activity concentration ratio which is found using the following equation:


$$RMBLR=\frac{{\left[A\right]}_{RM}}{{\left[A\right]}_{BL}}=\frac{RMECFF}{1-HCT}$$

[A]_RM_, [A]_BL_ = Activity concentrations in red marrow and blood

RMECFF = The red marrow extracellular fluid fraction is assumed to have a constant value of 0.19 [12]. It should be noted that this value was obtained from a study of the albumin space in the red marrow of rabbit femur and may not be appropriate for patients whose marrow has been compromised (eg, by previous therapy). For these patients, an RMBLR of 0.32 may be more appropriate [13] and used for conservatism.

HCT = Hematocrit of the patient

C _blood_ = Cumulative activity concentration in blood per administered activity (µCi-h/mL/mCi).

This is calculated by determining the area under the curve (AUC) of blood concentration per administered activity (µCi/mL/mCi) over time (hours). An Excel spreadsheet was used to determine the AUC from 0 to 48 hours postdose. The AUC for beyond the last time point (AUC 48 h → ∞) is calculated by determining the terminal elimination rate (λ) between the last 2 time points (ie, 24 and 48 hours) and then dividing the blood concentration per administered activity value at 48 hours by the λ (this is simply the integration of 48 hours to ∞ for an exponential function). An Excel spreadsheet was used to perform this calculation. The total C blood is found by adding the AUC 0 → 48 h and the AUC 48 h.

${\tilde{A}}_{TB}$ = Cumulative activity of the total body per administered activity (µCi-h/mCi). This is calculated by determining the AUC of activity (µCi) in the total body (as determined from the uptake probe measurement or gamma camera assessment) per mCi administered over time (hours). An Excel spreadsheet was used to determine the AUC from 0 to 48 hours postdose. The AUC for beyond the last time point (AUC 48 h → ∞) is calculated by determining the terminal elimination rate (λ) between the last 2 time points (ie, 24 and 48 hours) and then dividing the total body activity per administered activity at 48 hours by the λ (this is simply the integration of 48 hours to ∞ for an exponential function). An Excel spreadsheet was used to perform this calculation. The total ${\tilde{A}}_{TB}$ is found by adding the AUC 0 → 48 h and the AUC 48 h → ∞. m_TB_ = Mass of patient (g).

### Statistical Analysis

Dosimetry calculations were assessed for the ratio to blood activity and cumulative activity concentration, maximal and terminal effective doses, and fraction of administered dose. The cohort was evaluated for differences in clinicopathologic variables and RAI dosimetry calculations by preparation type. Using Mann-Whitney *U* and Kruskal Wallis tests, categorical variables were compared using Fisher exact and continuous variables. Multivariable regression analyses adjusting for age and AJCC stage were conducted to compare percentage reduction in Tg in the rhTSH vs THW groups. Data analysis was completed with STATA version 17 (Stata Corp LLC, College Station, TX).

## Results

In total, 51 patients received 55 I-131 treatments using dosimetry to calculate the maximum allowable treatment dose: 22 (40%) by rhTSH and 33 (60%) by THW. In this cohort, 4 patients received dosimetry-guided RAI twice, 3 of which were prepared with THW and 1 with rhTSH on repeat treatment. Thirty (55%) of the entire group were female. The majority (86.8%) were White, and papillary was the most common type of thyroid carcinoma (61.8%).

Race, sex, body mass index, size of tumor, baseline creatinine, glomerular filtration rate, and platelets were not statistically different between the rhTSH and THW groups. There were significant differences in histologic types of thyroid cancer between the therapeutic groups: 72.73% of the THW group, compared to 45.45% of the rhTSH, had papillary thyroid carcinoma, and 22.73% of the rhTSH group had follicular thyroid carcinoma, compared to 3% of the THW group (*P* = .0405). There was a significant difference in staging between the groups, using both 7th (*P* = .001) and 8th (*P* = .009) edition AJCC staging showing more stage 3 and 4, as well as more metastatic cases in rhTSH compared to the THW group (Table 1).

**Table 1.** Categorical variables baseline prior to iodine-131 therapy with dosimetry

	rhTSH (n = 22)	THW (n = 33)	*P*
Female	11 (50%)	19 (57.58%)	.595
Number of RAI treatments^*a*^			
1	8 (36.36%)	1 (3.03%)	.048
2	7 (31.82%)	18 (54.55%)	
3	6 (27.27%)	12 (36.36%)	
4	1 (4.55%)	2 (6.06%)	
Race			.951
Asian	2 (9.09%)	3 (9.68%)	
Black	1 (4.55%)	1 (3.23%)	
White	19 (86.36%)	27 (87.10%)	
Histology			.041
Classic papillary	11 (45.45%)	24 (72.73%)	
Follicular-variant papillary	3 (13.64%)	4 (12.12%)	
Tall cell-variant papillary	0 (0%)	1 (3.03%)	
Diffuse sclerosing-variant papillary	1 (4.55%)	1 (3.03%)	
Follicular	5 (22.73%)	1 (3.03%)	
Oncocytic	1 (4.55%)	2 (6.06%)	
Poorly differentiated	1 (4.55%)	0 (0%)	
Posttreatment iodine-131 uptake			
Thyroid bed	8 (38.10%)	12 (36.36%)	1.0
Cervical LN	1 (4.76%)	1 (3.03%)	1.0
Lung	11 (52.38%)	14 (42.42%)	.579
Bones	8 (38.10%)	7 (21.21%)	.220
Other	1 (4.76%)	9 (27.27%)	.069
N stage			.8775
0	6 (30%)	6 (18.75%)	
1a	1 (5%)	6 (18.75%)	
1b	13 (65%)	20 (62.5%)	
M stage			.066
0	3 (14.29%)	13 (40.2%)	
1	18 (85.71%)	19 (59.38%)	
8th edition AJCC stage			.009
1	1 (4.76%)	9 (27.27%)	
2	3 (14.29%)	12 (36.36%)	
3	0 (0%)	1 (3.03%)	
4a	1 (4.76%)	1 (3.03%)	
4b	16 (76.19%)	10 (30.30%)	
Location of metastases at treatment			
Lung	17 (80.95%)	17 (51.52%)	.043
Bone	10 (47.62%)	5 (16.13%)	.027
Brain	1 (4.76%)	0 (0%)	.404

Abbreviations: AJCC, American Joint Cancer Commission; LN, lymph node; RAI, radioiodine; rhTSH, recombinant human TSH; THW, thyroid hormone withdrawal.

^a^4 patients received dosimetry-guided RAI twice, 3 of which were prepared with THW and 1 with rhTSH on repeat treatment. If < 5, we used the Fisher exact (not 1-sided) model. If more than 2 groups: Kruskal Wallis: chi^2^ with ties.

The rhTSH group was significantly older than the THW group, with a median (interquartile range) age of 69 years (60.2-74.6) in the rhTSH group compared with 49.13 years (37.2-63.79) in the THW group (*P* = .0008). The rhTSH group had a higher median TSH (158.07 vs 79.6; *P* = .0038) and a higher median stimulated Tg 790.5 vs 33.5; *P* = .0031) but a shorter follow-up duration than the THW group. Blood counts and tumor size at baseline were not different between the 2 groups (Table 2).

**Table 2.** Continuous variables for each group at baseline prior to iodine-131 with dosimetry

Variables, median (IQR)	rhTSH (n = 22)^*a*^	THW (n = 33)^*a*^	*P* value
Age (y)	69.0 (60.3-74.6)	49.13 (37.2-63.79)	.0008
Body mass index (kg/m^2^)	31.79 (26-37.9)	29 (24-31.6)	.2924
Size of tumor (cm)	2.8 (1.8–4)	2.8 (2-4.3)	.8383
Creatinine	1.03 (0.86-1.36)	1.15 (0.97-1.42)	.3908
Glomerular filtration rate (mL/min)	58 (50-60)	55 (46-60)	.7051
White blood cell count (cells/μL)	5.8 (4.8-7.4)	5.85 (5.05-7.15)	.8414
Hematocrit (L/L)	39.5 (36-41.5)	42.35 (39.15-45.05)	.0364
Platelet count (cells/μL)	228 (193-266)	203 (177-258)	.2372
Thyroglobulin (ng/mL)	790.5 (98.5-6000)	33.5 (11-161)	.0031
TSH (mIU/L)	158.07 (10 272-206.197)	79.6 (51.9-130.32)	.0038
Length of follow-up (days)	68 (8.5-355.5)	2655 (1198-3785)	.0000

Abbreviations: I-131, iodine-131; IQR, interquartile range; rhTSH, recombinant human TSH; THW, thyroid hormone withdrawal.

^a^4 patients received dosimetry-guided RAI twice, 3 of which were prepared with THW and 1 with rhTSH on repeat treatment.

The cumulative activity of the total body per administered activity was significantly higher in the rhTSH group (*P* = .046), making the maximum allowable dose for retention of 80 mCi to a lung metastasis lower in the rhTSH group (*P* = .020). The terminal effective half-life at 24 to 48 hours in the whole body was longer in the rhTSH group compared to the THW group (21.9 vs 17.1 hours; *P* = .014). When analyzed only in the n = 37 distant metastatic cases, terminal effective half-life at 24 to 48 hours in the whole body was still significantly longer in the rhTSH than in the TWH group (20.3 vs 17.6 hours; *P* = .046). Although the terminal effective half-life at 24 to 48 hours in the red marrow was longer in the rhTSH group compared to the THW group (19.4 vs 15.3 hours; *P* = .029), this was not significantly different when only analyzed in the n = 37 distant metastatic cases (18.8 vs 15.3 hours; *P* = .078). The permissible RAI dose calculated by dosimetry was lower in the rhTSH compared to the THW group (187.5 mCi vs 259.9 mCi; *P* = .0000) (Table 3).

**Table 3.** Dosimetry calculations for iodine-131 therapy

Dosimetry calculations, median (IQR)	rhTSH (n = 22)^*a*^	THW (n = 33)^*a*^	*P*
Red marrow to blood activity concentration ratio	0.51 (0.49-0.54)	0.52 (0.362-0.56)	.7899
Cumulative activity concentration in blood per administered activity (mCi)	0.914 (0.717-1.351)	0.925 (0.74-1.087)	.6991
The cumulative activity of the total body per administered activity (mCi)	33 725 (25 652-110 676)	26 443 (20 886-31 488)	.0463
Maximum dose to give whole-body retention of 80 mCi (if lung met)	357.5 (199-651)	651 (397-926)	.0204
Maximum dose to give an absorbed dose of 200 rad to marrow (mCi)	583.5 (397-884)	619 (487-914)	.5707
Maximum dose to give an absorbed dose of 300 rad to the marrow (mCi)	—	275 (240-283)	—
Terminal effective half-life whole body 0-24 hours	22.25 (16-38.43)	17.2 (14.2-20.52)	.0782
Terminal effective half-life whole body 24-48 hours	21.86 (17.77-35.419)	17.133 (13.32-20.789)	.0144
Terminal effective half-life whole body 24-48 hours among distant metastatic cases (n = 37)	20.3 (17.77-30.23)	17.57 (14.27-20.79)	.0465
Fraction of administered dose whole body at 24 hours	0.5115 (0.374-0.682)	0.424 (0.331-0.483)	.0768
Fraction of administered dose whole body at 48 hours	0.2535 (0.151-0.403)	0.16 (0.108-0.211)	.0250
Residence time whole body (hours)	33.725 (25.65-47.88)	25.87 (22.22-31.4)	.0463
Terminal effective half-life red marrow hours 0-24 hours	18.29 (13.63-33.49)	14.68 (11.39-18.41)	.0717
Terminal effective half-life red marrow hours 24-48 hours	19.445 (15.76-37.89)	15.33 (11.9-20.3)	.0291
Terminal effective half-life red marrow hours 24-48 hours among distant metastatic cases (n = 37)	18.845 (15.76-26.85)	15.33 (11.9-20.3)	.0780
Fraction of activity in bone marrow at 24 hours	0.45 (0.32-0.54)	0.38 (0.32-0.47)	.3746
Fraction of activity in bone marrow at 48 hours	0.2 (0.12-0.3)	0.13 (0.08-0.21)	.0621
Residence time red marrow (hours)	1.55 (1.11-2.36)	1.64 (1.2-3.3)	.4444
RM dose to the patient (REM)	58.89 (44.33-87.28)	75.53 (57.3-101.73)	.1747
RAI dose (mCi)	187.5 (167-197.9)	259.9 (202-306)	.0000

Abbreviations: I-131, iodine-131; IQR, interquartile range; RAI, radioiodine; rhTSH, recombinant human TSH; RM, rad marrow; THW, thyroid hormone withdrawal.

^a^4 patients received dosimetry-guided RAI twice, 3 of which were prepared with THW and 1 with rhTSH on repeat treatment.

As for the response to RAI treatment, creatinine, white blood cells, HCT, and platelets were similar between groups 3 months posttreatment. The percentage reduction in Tg at 3 months posttreatment was not statistically different between groups on univariate analysis, and after adjusting for age and AJCC stage (Table 4).

**Table 4.** Laboratory results 3 months following iodine-131 with dosimetry

Laboratory parameters	rhTSH (n = 22)	THW (n = 33)	*P*
Creatinine (mg/dL)	0.99 (0.81-1.47)	0.85 (0.69-1)	.0757
Glomerular filtration rate (mL/min)	60 (56-60)	60 (60-60)	.0479
White blood cell count (cells/μL)	7.1 (4-10.4)	5.3 (4.2-5.9)	.1695
Hematocrit (L/L)	36.2 (33.4-38.7)	39.55 (36.15-40.9)	.1450
Platelet count (cells/μL)	183 (144-221)	186 (157-246.5)	.3337
TSH (mIU/L)	0.5325 (0.248-2.22)	0.175 (0.0515-0.63)	.0682
Thyroglobulin*^a^* (ng/mL)	129 (5.2-437)	2.4 (.6-67)	.0585
Thyroglobulin percentage reduction (%)	76.8 (35.9-91.3)	91.4 (58.9-96.9)	.1212

Abbreviations: rhTSH, recombinant human TSH; THW, thyroid hormone withdrawal.

^a^Data not available for n = 12.

## Discussion

In this study, we demonstrate the efficacy and safety of rhTSH compared with THW among patients with metastatic DTC treated with dosimetry-guided I-131 therapy. We observed a similar reduction in Tg after 3 months and no difference in changes in white blood cells after I-131 therapy in both groups of patients. A strength of our study is focusing on only dosimetry-guided I-131 therapy to homogeneously compare the performance and safety of rhTSH and THW in metastatic DTC.

Patients that are older or with comorbidities should avoid untoward effects of hypothyroidism, which could explain the older age in the rhTSH group compared to the THW group in our study, similar to prior studies that reported rhTSH to be safe and successful for inducing I-131 uptake in local and metastatic DTC based on the Thyrogen Compassionate Use Program [5-9]. Despite the older age in the rhTSH group in our study, the similar biochemical response and tolerability compared to THW shows that rhTSH may be superior for achieving outcomes as older age is a known risk factor for poor prognosis in metastatic DTC.

The frequencies of stage IV DTC and distant metastases were higher in the rhTSH group than the THW group, despite both groups demonstrating equivalent percentage reduction in Tg 3 months after I-131 therapy. This shows that rhTSH preparation was similar or even better for biochemical response to treatment. These findings of the efficacy of rhTSH are corroborated by recent studies in patients with metastatic DTC where preparation with rhTSH achieved comparable benefits of RAI therapy as those treated after THW [8, 14]. Hence, the concern for potentially less optimal response because of lower RAI uptake in metastatic DTC lesions after rhTSH compared to THW reported in an older study [10] was not seen in our cohort. Although Klubo-Gwiezdzinska et al [8] also evaluated the structural responses to RAI treatment, our study focused on analyzing the dosimetry performance of rhTSH compared to THW in preparing patients for dosimetry-guided I-131 therapy. Additionally, the follow-up duration of subjects in the 2 groups was different. Hence, we have not evaluated the structural response to RAI therapy, as that was not what our study was designed to evaluate.

As for the I-131 dosimetry metrics, the terminal effective half-life of I-131 was longer in the rhTSH than the THW group; however, when only examining the patients with metastatic DTC, the difference was less significant in the whole body and not present in the red marrow. Remy et al reported the mean effective half-life to be shorter by 31% in the rhTSH group (10.5 hours) compared to the THW group (15.7 hours) [4]. However, other studies have shown a higher effective half-life of RAI with rhTSH than THW, but not all patients treated in these studies had distant metastasis [10, 15]. We attribute this dissimilarity in the studies’ findings to the differences in the proportion of distant metastatic cases in the rhTSH compared to the THW groups. In our study, the cumulative median I-131 dose was lower in the rhTSH group compared to THW, but clinical judgment superseded the calculated allowable dosage. This was also demonstrated in a systematic review by Ma et al of 4 randomized controlled clinical trials comparing rhTSH and THW methods for remnant ablation in patients with DTC [16]. However, that study had limited data on patients with metastatic DTC. Overall, the lower I-131 permissible dose calculated by dosimetry in the rhTSH group demonstrates that the long-term risk of toxicities could be lower with this as compared to the THW group. Multiple prior studies have also demonstrated that a lower I-131 dose is calculated in the rhTSH group [4, 15, 17, 18].

As for short-term toxicities, blood counts were similar between the 2 groups 3 months posttreatment. Other studies have demonstrated no significant difference in the rates of not only hematologic toxicities but also xerostomia and restrictive pulmonary disease when comparing rhTSH to THW [8, 14]. Hence, our results and those from prior studies demonstrate the comparable safety of rhTSH in the I-131 treatment of metastatic DTC.

Our study has limitations in that it does not evaluate longer term structural and biochemical responses in the 2 groups, as demonstrated by other studies [8, 14]. We could not report this comparison because the follow-up durations were dissimilar between the 2 groups. However, since a similar effect on response and toxicity has been shown previously [8, 14], our focus was to evaluate how these 2 regimens perform during dosimetry for I-131 treatment. Additionally, the retrospective design of our study is affected by potential confounders that could impact treatment efficacy. We also did not have a large enough sample to adjust for factors that were different between the 2 groups besides age and stage. However, adjusting for these 2 factors still showed a similar percentage reduction in Tg in the 2 groups.

Our study also did not address the difference in quality of life between the 2 groups. Other studies have demonstrated that the rhTSH method was associated with better quality of life, fewer hypothyroid symptoms, and improved mental health and functional status [19-22]. It can be expected that the absence of hypothyroidism and faster clearance of RAI from the body could account for this. The lower total body, bone marrow, and gastrointestinal radiation exposure for a given administered activity by the rhTSH method compared to THW, as shown in our study and other studies [4, 17, 18], could also reduce the likelihood of long-term toxicities from I-131, which do influence the quality of life. However, these data need further confirmation.

In conclusion, we demonstrate that rhTSH is noninferior to THW as a preparation method for dosimetry-guided I-131 treatment both in terms of short-term efficacy and safety. Hence, rhTSH can be safely administered to patients who cannot tolerate THW, potentially resulting in a lower cumulative RAI dose. Further research with a larger sample and longer follow-up is necessary to validate the clinical significance of these findings.

## Data Availability

Some or all datasets generated during and/or analyzed during the current study are not publicly available but are available from the corresponding author on reasonable request.

## Abbreviations

AJCC: American Joint Cancer Commission
AUC: area under the curve
DTC: differentiated thyroid cancer
I-131: iodine-131
RAI: radioiodine
RBM: red bone marrow
Tg: thyroglobulin
rhTSH: recombinant human TSH
THW: thyroid hormone withdrawal
